# Congenital Nemaline Myopathy in Two Neonates With Different Mutations: A Case Series and Literature Review

**DOI:** 10.7759/cureus.45197

**Published:** 2023-09-13

**Authors:** Farzeen Mohtisham, Maram Althagafi, Aiman Shawli, Adel Sallam

**Affiliations:** 1 Neonatology, King Saud Bin Abdulaziz University for Health Sciences, Jeddah, SAU; 2 Neonatology, King Abdulaziz Medical City, Ministry of National Guard Health Affairs, Jeddah, SAU; 3 Pediatrics, King Abdulaziz Medical City, Ministry of National Guard Health Affairs, Jeddah, SAU

**Keywords:** phenotype, genotype, neonatal, myopathy, inherited, congenital

## Abstract

Nemaline myopathy is a skeletal muscle disorder characterized by a wide range of severity and variable presentation. While most cases present in the neonatal period with symptoms, such as hypotonia, muscle weakness, and respiratory insufficiency, delayed onset in childhood or adulthood is also observed. The pathogenesis of nemaline myopathy involves at least 12 genes, and the condition can arise from de novo mutations or be inherited in a dominant or recessive manner. In this study, we present two cases of neonates admitted to a neonatal intensive care unit (NICU) exhibiting hypotonia, muscle weakness, and respiratory insufficiency. Both cases were diagnosed with congenital nemaline myopathy, with each patient displaying distinct mutations. This report highlights the clinical and genetic heterogeneity of this condition, emphasizing the importance of early recognition and genetic evaluation for accurate diagnosis and appropriate management of affected individuals.

## Introduction

Nemaline myopathy is a skeletal muscle disorder with muscle weakness of variable severity. It presents in the neonatal period in most cases with hypotonia and muscle weakness, but delayed presentation in childhood or adulthood is also recognized [1]. At least 12 genes are involved in the pathogenesis of nemaline myopathy, which can be caused by de novo mutation or can be inherited dominantly or excessively [1]. It is very rare with an incidence of 1:50,000 live births [2]. 

Here, we report two cases of neonates who were admitted to our neonatal intensive care unit (NICU) due to hypotonia, muscle weakness, and respiratory insufficiency and diagnosed to have congenital nemaline myopathy with different mutations and clinical picture. To our knowledge, these are the first reported cases from Saudi Arabia.

## Case presentation

Case 1

A baby girl was first born to a Saudi couple with positive second-degree consanguinity. The mother and father were 27 years old and 29 years old, respectively, and were healthy. There was no family history of neonatal death and metabolic or neuromuscular diseases. Her antenatal history including ultrasound was normal. She came in labor at 32 weeks and was delivered by emergency cesarean section due to breech presentation and abnormal cardiotocograph (CTG). The baby was born with no spontaneous movements, apneic with bradycardia, and required immediate resuscitation that ended by endotracheal intubation. The Apgar scores were 2 at one minute, 2 at five minutes, and 5 at 10 minutes. Her birth weight, head circumference, and length were all below the fifth percentile (1.460 kg, 21 cm, and 42 cm, respectively). She was transferred to the NICU and connected to mechanical ventilation.

She had profound hypotonia with a frog-like position and complete absence of movements and tendon reflexes in all four limbs. There were multiple bruises on the trunk and lower limbs, and X-ray showed right femur fracture. She looked dysmorphic with prominent forehead, low-set ears, hypertelorism, flat nasal bridge, fish mouth, micrognathia, and arachnodactyly. She had bilateral lagophthalmos and absent corneal reflexes with incomplete eye closure, which eventually resulted in bilateral exposure keratitis despite using local lubricant therapy. The pupils were equal bilaterally with sluggish reaction to the light. She had an open-mouth posture with decreased gag reflex and absent swallowing function necessitating nasogastric tube feeding. She continued to have poor respiratory effort, which required tracheostomy and continued ventilator support.

She was managed by a multidisciplinary team involving pediatric neurology, orthopedic, physiotherapy, genetic, and metabolic teams. All routine laboratory investigations were normal, including complete blood count; renal, liver, and thyroid functions; glucose; creatinine kinase; lactic acid; ammonia; newborn screening; and metabolic workup.

**Figure 1:**
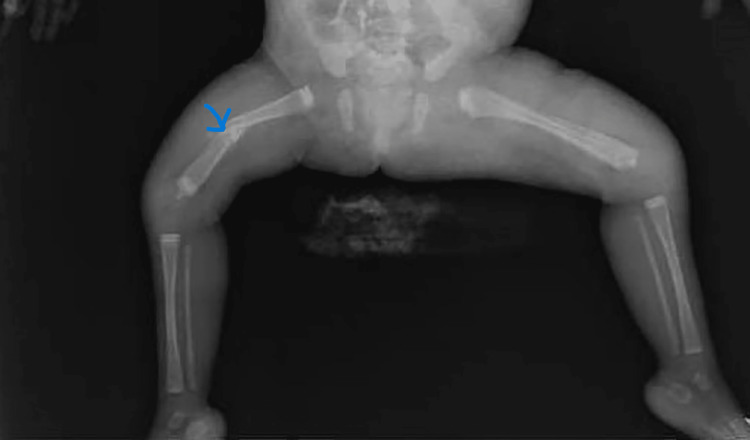
X-ray showing fracture of the right femur of case 1

Imaging of the heart and abdomen was normal. Skeletal survey showed generalized diffuse osteopenia with evidence of thin elongated bones with fracture of the right femur (Figure 1).

**Figure 2:**
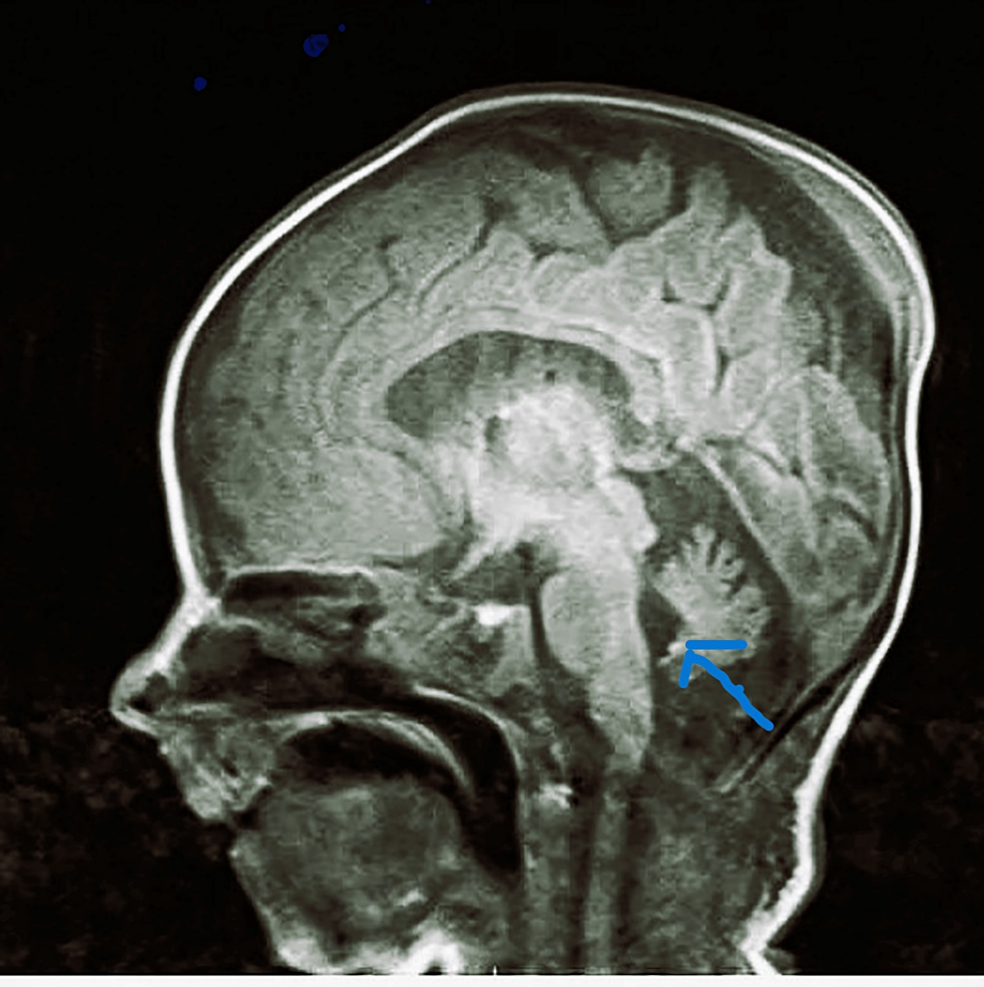
MRI brain of case 1

She developed a new fracture later on in the left humerus for which a splint was applied. Brain MRI showed bilateral ischemic foci involving the periventricular white matter and centrum semiovale and the cortex at the level of the left Rolandic sulcus with mild inferior vermian hypoplasia (usually not a presentation of myopathy) (Figure 2).

Electroencephalography showed evidence of mild to moderate bitemporal cerebral dysfunction and evidence of mild encephalopathy. The genetic testing revealed nemaline myopathy type 8 caused by a mutation in the KLHL40 gene. She was continued with ventilator support via tracheostomy and other supportive care until her death due to aspiration pneumonia around four months of age.

Case 2

A baby boy was born to a 28-year-old Saudi female, gravida 5, para 4, with a history of two previous infant deaths (born and managed at a different hospital) with hypotonia since birth. Both of them required oxygen and tube feeding, and they died within a few months of age due to respiratory failure without any established diagnosis.

The parents were first cousins with no other significant family history apart from one of their nephews who was diagnosed as a case of Prader-Willi syndrome and was being managed at another center.

He was born at 36 weeks with a birth weight of 2,490 grams, head circumference of 33.5 cm, and length of 50 cm (all parameters within normal centile for age). The Apgar scores were 7 at one minute and 8 at five minutes. He was started on nasal cannula oxygen to maintain good saturation.

**Figure 3:**
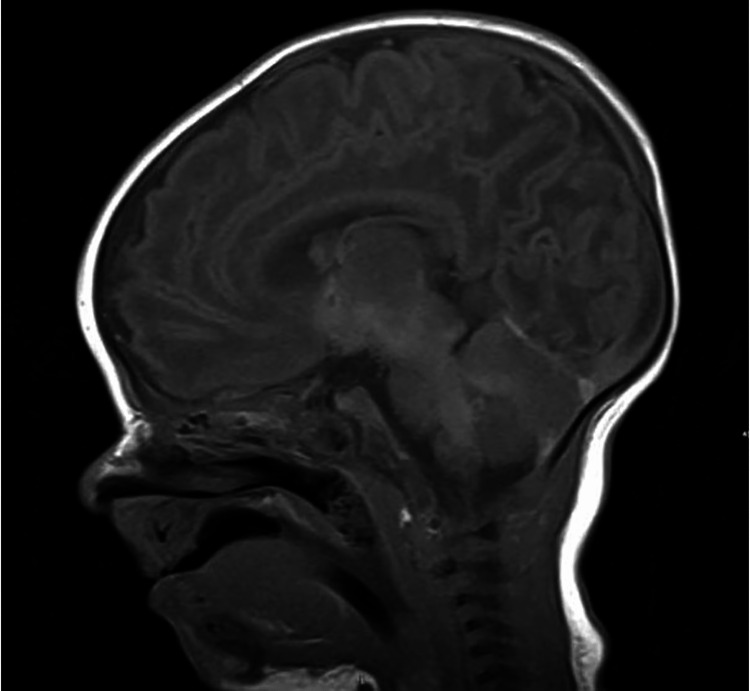
Normal MRI brain of case 2

He was noted to be hypotonic since birth with a frog-like position, weak Moro reflex, and myopathic facies. The workup for hypotonia revealed a normal renal and liver profile and a normal metabolic workup. Imaging by MRI showed a structurally normal brain (Figure 3).

The genetic study by whole exome sequencing showed a homozygous variant in the NEB gene (NM_001271208.2) with both parents being heterozygous for the same gene. He was continued on nasal cannula oxygen with tube feeding and had failure to thrive despite adequate nutrition and caloric adjustment. He continued to have worsening hypotonia and eventually died at four months of age due to respiratory failure.

## Discussion

Nemaline myopathy is a heterogeneous muscular disorder characterized by a wide range of clinical presentations, from neonatal death to mild limb weakness. It is defined by the presence of nemaline rod-like structures within skeletal muscle fibres, leading to severe weakness. The term "nemaline" was originally introduced by Shy and colleagues in 1963 to describe a distinctive nonprogressive myopathy characterized by thread-like structures within muscle fibers [3].

Clinically, nemaline myopathy encompasses six distinct types: (1) classic congenital type, which typically presents with hypotonic infants displaying facial and respiratory weakness; (2) severe congenital variant, which is characterized by a profound lack of movement and respiratory failure (contractures, fractures, and arthrogryposis multiplex have been associated with this variant (10-20%)); (3) intermediate congenital type, whose clinical manifestations appear later in childhood; (4) mild childhood or adolescent type, which presents in childhood and adolescence with mild proximal muscle weakness; (5) adult-onset type, whose clinical manifestations appear in adulthood as mild muscle weakness; (6) group of patients with variable features, including cardiomyopathy and ophthalmoplegia [3].

However, there is a new proposed classification taking into consideration the clinical picture and the genetic mutation [1]: (1) severe nemaline myopathy (with contractures or fractures at birth or with no respiratory effort or no movements at birth) (ACTA1, NEB, LMOD-3, KLHL40, KLHL41, TNNT3, TPM2, and TPM3); (2) congenital nemaline myopathy (with perinatal onset and milestones delayed but reached) (NEB, ACTA1, CFL-2, and TPM2); (3) mild (childhood or juvenile-onset) nemaline myopathy (ACTA1, NEB, TPM2, TPM3, KBTBD13, MYPN, and dominant mutations in TNNT1); (4) recessive TNNT1 (Amish) nemaline myopathy; and (5) childhood-onset nemaline myopathy with slowness of movements and core-rod histology (KBTBD13).

Nemaline myopathy type 8 is an autosomal recessive disorder resulting from homozygous pathogenic variants in the KLHL40 gene (OMIM#615348). This type is characterized by severe neonatal muscle weakness, akinesia or hypokinesia, contractures, facial muscle involvement, respiratory insufficiency, skeletal fractures, and swallowing difficulties, which typically manifest in the early neonatal period [4]. In our report, the clinical features of our first case were consistent with previous reports, including myopathic facies, severe muscle weakness, contractures, fractures at birth, and absent swallowing function. However, she also exhibited additional features not reported in previous cases, such as mild encephalopathy on electroencephalography and bilateral ophthalmoplegia, resulting in bilateral exposure keratitis despite local lubricant therapy.

It is noteworthy that nemaline myopathy involves mutations in at least 12 genes. The most common forms are associated with mutations in the ACTA1 and NEB genes, encoding skeletal muscle α-actin and nebulin, respectively [1].

In the baby in case 1, the whole exome sequence showed homozygous duplication variant c.118_203dup.p (Gly70Serfs*23) (chr 3:42727224;hg 19) in the KLHL40 gene, resulting in a frameshift that results in a premature stop codon and subsequent mRNA degradation or truncation of the protein. Parallel analysis of the parents revealed that both parents are heterozygous for the variant detected. Pathogenic variant in the KLHL40 gene causes autosomal recessive nemaline myopathy type 8 (OMIM 615340) [5].

Pathogenic variants for the NEB gene (NM_0012712082.2) are known to cause autosomal recessive nemaline myopathy type 2 (OMIM 256030). This is also known to present in the early neonatal period with variable presentation ranging from mild to severe myopathy. As in our second case, the whole exome sequence showed homozygous duplication variant c.24533dup p.(Leu8179Phefs18l) in the NEB gene (OMIM 161650) (chr2: 152352847), resulting in a frameshift that results in a premature stop codon and subsequent mRNA degradation or truncation of the protein. Parallel analysis of both parents revealed that they are heterozygous for the variant detected. Considering the clinical presentation and family history of infant deaths and the genetic mutation, it is consistent with classic neonatal congenital myopathy (Table 1).

**Table 1:** Genotype and phenotypic presentations of our cases

	Gene	Time of presentation	Eye involvement	Respiratory failure	Swallowing	MRI brain
Case 1	KLHL40	Profound muscle weakness since birth	Lagophthalmos with exposure keratitis	Severe, mechanically ventilated since birth	Severely affected	Focal changes
Case 2	NEB	Mild hypotonia at birth > distal muscles worsened with time	None	Mild initially, gradually worse with time	Significantly affected	Structurally normal

In both cases, the parents were extensively counseled by the genetic and neonatology team about the prognosis of the disease, nature of inheritance, and possibility of recurrence in future pregnancies. They were also given the option of future pregnancy via in vitro fertilization and preimplantation genetic diagnosis to avoid the trauma of having similar offspring.

Muscle biopsy is diagnostic for nemaline myopathy based on the presence of pathognomonic nemaline rod-like bodies in skeletal muscle fibers. However, it is not easy to perform due to various factors, including the patient's size and clinical condition. Moreover, with the development of genetic testing, muscle biopsies are no longer the gold standard [6]. Our patients did not undergo muscle biopsy because the parents refused consent for this invasive procedure especially when the diagnosis was already established by genetic diagnosis.

No effective treatment is currently available for nemaline myopathy patients, although one patient with KLHL40-related nemaline myopathy was reported to have had a dramatic improvement after treatment with acetylcholinesterase inhibitors [7].

Supportive management including orogastric/nasogastric tube or gastrostomy tube feeding should be considered in patients with recurrent aspiration and swallowing difficulties. Mechanical ventilation may be required if the patient has respiratory failure [8-9]. Our first patient was ventilator dependent since birth and required nasogastric feeding tube throughout her hospital stay as no swallowing function was identified. The second baby was managed on nasal cannula oxygen and tube feeding in addition to other supportive measures to minimize aspiration, such as suctioning and nebulizers.

## Conclusions

Congenital myopathy is a complex and diverse group of genetic disorders that affect muscle function from birth. This condition presents with a wide range of symptoms, including muscle weakness, hypotonia, and impaired motor development. While advancements in medical research have provided a better understanding of the underlying genetic mutations and molecular mechanisms involved in congenital myopathy, treatment options remain limited, and the primary focus is on symptomatic treatment and improving the quality of life.

We encourage healthcare workers to consider comprehensive workup for congenital myopathy in any infant with severe hypotonia, a family history of affected babies, contractures, and fragile bones. We also recommend genetic testing for the patients and their parents for diagnostic and prognostic purposes and for appropriate genetic counseling. In addition, parents can be offered assisted conception techniques with preimplantation genetic diagnosis for future pregnancies when indicated and feasible.

With continued efforts, there is hope for improved diagnostic accuracy, better management strategies, and potential breakthroughs in treatments for individuals living with congenital myopathy.
